# Exposure to 50 Hz electromagnetic field changes the efficiency of the scorpion alpha toxin

**DOI:** 10.1186/s40409-015-0040-9

**Published:** 2015-09-30

**Authors:** Milena Jankowska, Agnieszka Pawlowska-Mainville, Maria Stankiewicz, Justyna Rogalska, Joanna Wyszkowska

**Affiliations:** Nicolaus Copernicus University, Faculty of Biology and Environmental Protection, Torun, Poland; Department of First Nations Studies, University of Northern British Columbia, Prince George, Canada

**Keywords:** 50 Hz electromagnetic field, Alpha scorpion toxin, Cockroach, Bioelectrical activity

## Abstract

**Background:**

Extremely low-frequency (50 Hz) electromagnetic field (ELF-EMF) is produced by electric power transmission lines and electronic devices of everyday use. Some phenomena are proposed as “first effects” of ELF-EMF: the discrete changes in the membrane potential and the increase of the calcium channel activity as well as the intracellular concentration of Ca^2+^. Interaction of the scorpion alpha toxin with the sodium channel depends on the orientation of the charges and may be perturbed by changes in the membrane polarization. The toxin induces overexcitability in the nervous system and an increase in the neurotransmitters released with different consequences, mainly the paralysis of muscles. We assumed that the exposure to ELF-EMF 0.7 mT will change the effects of the insect selective scorpion alpha toxin (recombinant LqhαIT from *Leiurus quinquestriatus hebraeus*) at the level of the cercal nerve function, the synaptic transmission and on the level of entire insect organism. Taking into account the compensatory mechanisms in organisms, we tested in addition ten times higher ELF-EMF on whole insects.

**Methods:**

Experiments were performed *in vivo* on cockroaches (*Periplaneta americana*) and *in vitro* – on isolated cockroach abdominal nerve cord with cerci. In biotests, the effects of LqhαIT (10^−8^ M) were estimated on the basis of the insect ability to turn back from dorsal to ventral side. Three groups were compared: the control one and the two exposed to ELF-EMF – 0.7 and 7 mT. Bioelectrical activity of the cercal nerve and of the connective nerve that leaves the terminal abdominal ganglion was recorded using extracellular electrodes. LqhαIT (5 × 10^−8^ M) induced modifications of neuronal activity that were observed in the control cockroach preparations and in the ones exposed to ELF-EMF (0.7 mT). The exposure to ELF-EMF was carried out using coils with a size appropriate to the examined objects.

**Results:**

The exposure to ELF-EMF (0.7 mT) modified the effects of LqhαIT (5 × 10^−8^ M) on activity of the cercal nerve and of the connective nerve. We observed a decrease of the toxin effect on the cercal nerve activity, but the toxic effect of LqhαIT on the connective nerve was increased. Biotests showed that toxicity of LqhαIT (10^−8^ M) on cockroaches was reduced by the exposure to ELF-EMF (0.7 and 7 mT).

**Conclusions:**

The exposure to 50 Hz ELF-EMF modified the mode of action of the anti-insect scorpion alpha toxin LqhαIT at cellular level of the cockroach nervous system and in biotests. Toxin appeared as a usefull tool in distinguishing between the primary and the secondary effects of ELF-EMF.

## Background

Humans in highly industrialized countries are more and more exposed to extremely low-frequency (50 Hz) electromagnetic field (ELF-EMF). This field is produced by electric power transmission lines and electronic devices that help us to live comfortably. However, increasing intensity of ELF-EMF raises questions about its influence on plant, animal and human organisms. Numerous experiments have been carried out to clarify the problem of EMF effects. There is much evidence that even the exposure to ELF-EMF of very low intensity may alter molecular and cellular processes, as well as behaviour of animals [1–5].

Mechanisms of this influence have not been clarified to date and there is still the need for more research to develop our knowledge of “primary targets” of ELF-EMF. Some phenomena can be proposed as the “first” effects of an electromagnetic field in an organism: (1) discrete changes in the membrane potential induced by ELF-EMF energy; (2) increase of the calcium channel activity and in the intracellular concentration of Ca^2+^; (3) the enzymatic activity modification by mimicking the receptor binding [6–12]. The aim of our study was to estimate the influence of ELF-EMF exposure on the effects of the scorpion alpha toxin LqhαIT using the cockroach model to assess the response of the organism (*in vivo*) and to measure the nervous system bioelectrical activity (*in vitro*).

Scorpion alpha toxin LqhαIT (from *Leiurus quinquestriatus hebraeus*) is a polipeptide highly active on insects. It binds, as other scorpion alpha toxins, to the receptor site 3 on the voltage-dependent sodium channel and inhibits its inactivation [13, 14]. Sodium channels are responsible for the depolarizing phase of action potentials. Rapid inactivation of sodium inward current is the main factor responsible for the short action potential duration [15]. In the cockroach giant axon, the toxin LqhαIT extended the action potentials up to 500 folds and resulted in the generation of *plateau* action potentials [13, 16]. Moreover, after a single stimulation, LqhαIT induced several action potentials in the cockroach nerve cord instead of one [17]. Experiments on synaptic transmission in house fly muscles showed that LqhαIT caused a significant increase in excitatory junctional potentials amplitude [18]. Hyperexcitation of the nervous system and of the muscles caused progressive paralysis – a typical effect of scorpion alpha toxins [13, 16].

Scorpion alpha toxin receptor site 3 on sodium channel was defined as amino acid residues in extracellular linkers between segments S5-S6 in domain DI (“pore module”) and S3-S4 in DIV (“gating module”) [19]. Interaction of the alpha toxin with “the gating module” occurs by its “core domain” and with “the pore module” by “NC domain” [14, 20]. Attachment of “the core domain” prevents the normal outward movement of the positive charges in the DIV S4 segment during depolarization; it is trapped in its inward position, which results in uncoupling of fast inactivation from activation of the channel [21]. Affinity of scorpion alpha toxins to sodium channel is higher in its closed state. When the membrane potential is more negative than –80 mV, the association rate of the toxin with the channel does not depend on the potential; depolarization decreases binding of the toxin, depending on a group of alpha toxins [22]. All these facts clearly suggest that binding and efficiency of the scorpion alpha toxin may depend on the orientation and redistribution of the charges in the sodium channel and in the toxin.

An electromagnetic field (50 Hz) is a form of energy that may directly influence distribution of charges important for the toxin action. It is well known that even very weak external electric fields (1-5 V/m) and magnetic fields (near 50 μT) might modify bioelectrical activity of neurons by perturbation of the membrane potential and gating of the voltage-dependent channels [23–26]. Reference levels for occupational and general public exposure to the components of ELF-EMF are, respectively, electric fields of 10 kV/m, magnetic fields 0.5 mT, and electric fields of 5 kV/m, magnetic fields 0.1 mT which are values higher than those that can modify the bioelectric activity of a nervous system [27, 28].

The second process in which the effect of the alpha toxin may be modified by the ELF-EMF is the synaptic transmission. The exposure to the electromagnetic field may change the toxin effects on the postsynaptic side through the influence on calcium concentration in presynaptic terminals as well as in motoneurons, in this way, modifying the general toxin effect on the entire insect organism.

We assumed that the exposure to ELF-EMF (0.7 mT) may change the activity of the insect selective scorpion alpha toxin (LqhαIT) at the level of the cercal nerve function, the synaptic transmission and on the entire insect organism. The value of 0.7 mT has been chosen as the moderate level between the reference level for occupational exposure (0.5 mT) and the magnetic flux density 1 mT – one of the most frequently used field intensities in laboratory studies aimed at elucidating the biological effects of ELF-EMF [29]. There is also data indicating that some home appliances working at the same time in a small space can produce an electromagnetic filed reaching the intensity of 1 mT [30]. At the level of the whole organism, different compensatory mechanisms decrease the effects of environmental stress factors. Therefore, we also examined the influence of a ten-time higher ELF-EMF intensity (7 mT) on the toxin efficiency on the insect organism. A corresponding ELF-EMF intensity is used for example in magnetic field therapy [31].

## Methods

### Electrophysiological experiments

The experiments were performed on a male cockroach *Periplaneta americana* obtained from our own colony. The influence of the ELF-EMF exposure on the mode of action of the LqhαIT toxin have been tested *in vitro* on the escape system of a cockroach *P. americana*. For this purpose, we have used the experimental setup for extracellular recordings of the bioelectrical activity of ventral nerve cord described in detail in our previous papers [32, 33]. The preparation contained the presynaptic part (the cercal nerve) and the central postsynaptic one (the connective nerve).

Recombinant LqhαIT toxin (from *Leiurus qinquestriatus hebraeus*) has been purchased from Latoxan (France) and dissolved in physiological saline to 10^−8^ M and 5 × 10^−8^ M concentrations. The physiological saline contained: NaCl – 210 mM, KCl – 3.1 mM, CaCl_2_ – 5 mM, MgCl_2_ – 5.4 mM, pH = 7.2 was adjusted with Hepes – 5 mM (all chemicals were purchased from Sigma).

**Fig. 1:**
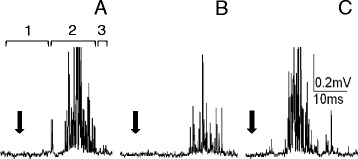
Extracellular recordings of cockroach nervous system bioelectrical activity. (**a**) An example of recordings of the cercal nerve activity as a response to cercus mechanostimulation (marked by black arrow); 1 – recording of a spontaneous activity, 2 – recording of a response to mechanostimulation, 3 – recording of the activity after stimulation. (**b**) An example of corresponding recordings from the connective nerve with the same value of stimulus as in **a**. (**c**) An example of recordings of the connective nerve response to a larger stimulus

Together with the cercal nerves linked to the cerci, the abdominal nerve cord was isolated from the body of an adult male cockroach. The slow perfusion of experimental chamber ensured stable and appropriate hydration of the nerve cord and the cercal nerves; however, the cerci were kept dry. Bioelectrical recordings were performed from the cercal nerve and one connective nerve leaving the terminal abdominal ganglion using modified professional extracellular electrode (from Alpha Omega Engineering LTD, Israel) (Fig. 1a, b, c). A reference nonpolarised electrode was placed in the vicinity of the terminal ganaglion. The electrodes were connected by a preamplifier with a compensatory amplifier which permitted the recording of extracellular bioelectrical signals.

The activity was recorded in “resting” conditions and after the cercus stimulation by an air puff (applied with 1 Hz frequency), sent by mechanostimulator (Fig. 1a, b, c). The air puff was produced by a movement of a loudspeaker membrane under a generator control. The stimulus was regulated to be a little bit stronger than the threshold value. The neuronal activity was observed on an oscilloscope; for further analysis, data were stored by a computer using a modified program Hammeg.

**Fig. 2:**
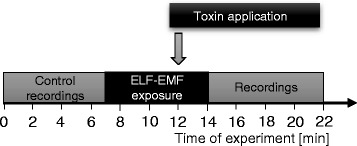
The scheme of a protocol of the electrophysiological experiments. In the first seven minutes of the experiment, the control activity of the cercal nerve or the connective nerve was recorded. In the next seven minutes, the nervous system of the insect was exposed to ELF-EMF. Two minutes before the end of the exposure, LqhαIT toxin was applied. After the end of the ELF-EMF exposure, the second part of the recordings was conducted

According to the protocol presented in Fig. 2, the size of the response to the stimulus, which we also called “bioelectrical activity” or “discharge frequency”, was estimated at the beginning of the experiment (control recordings) and after the exposure to the ELF-EMF. A simultaneous recording of the bioelectrical activity and the application of the electromagnetic field was impossible due to the electric noise. The ELF-EMF exposure was carried out for seven minutes, LqhαIT toxin (5 × 10^−8^ M) was added in the 5^th^ minute and the exposure was continued for another two minutes. Such protocol ensured the application of the toxin to the insect nervous system that had already been modified by the ELF-EMF exposure. In a previous series of experiments, we observed the first effect of LqhaαIT (5 × 10^−8^ M) application after two minutes – in corresponding time the recordings were re-started after the ELF-EMF exposure. In experiments without exposure to the ELF-EMF, to keep corresponding conditions, the stimulation and recordings were stopped for seven minutes – in time corresponding to the exposure period. In the control experiments, physiological saline was applied instead of the toxin.

### Setup for the ELF-EMF exposure of an isolated cockroach nerve cord

The coil (7 cm in diameter × 2 cm) used in this part of the research allowed us to insert it in the electrophysiological setup. The coil produced a 50 Hz homogenous (the maximum nonuniformity 16 %) magnetic field of 0.7 mT intensity and was installed around the experimental chamber in which the nerve cord was placed. No changes in temperature (measured with a thermocouple) were observed during the exposure.

### Toxicity tests

Male cockroaches were assigned into three groups: (1) sham – the control group, n = 15; (2) the group exposed to 50 Hz electromagnetic field of 0.7 mT intensity, n = 15; and (3) the group exposed to ELF-EMF of 7 mT intensity, n = 15. The effect of the LqhαIT toxin (10^−8^ M) on the insects exposed to the ELF-EMF of both intensities was observed. The concentration of the toxin was previously estimated to be a sublethal dose.

**Fig. 3:**
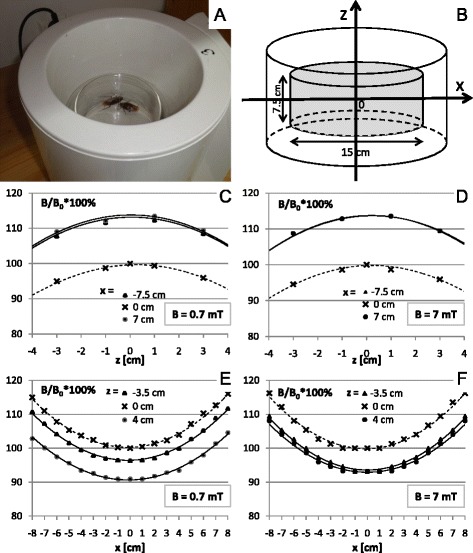
The setup for the exposure of a cockroach to an electromagnetic field. (**a**) Insects in the coil. (**b**) Coil coordinates system. (**c**-**f**) The magnetic flux density distribution in the coil along z axis (**c**, **d**) and x axis (**e**, **f**) for 0.7 mT and 7 mT (coordinates as at **b**)

At the beginning of each experiment, the insects were injected through thoracic inter-segmental membrane with 5 μL of the LqhαIT toxin solution using a Hamilton syringe. Just after the toxin injection, the insects were placed in glass chambers for the ELF-EMF exposure (Fig. 3a) or for the sham exposure. The toxin-induced contraction paralysis and its level have been evaluated as the insect’s ability to turn back from its dorsal to ventral side. This behavior was determined using a scale from 0 to 4, in which 4 corresponded to the highest ability to turn over, 1 when the insects were almost unable to move. The insects were placed on their dorsal sides on a special platform where the cockroaches that had not been injected with the toxin could easily turn back from the ventral to the dorsal side. The observations of the control and the ELF-EMF exposed insect behavior were done 1, 2 and 24 h after the toxin injection.

### The setup for the exposure of the insects to the ELF-EMF

An electromagnetic field (with the domination of the magnetic component) was generated by a coil wound around a plastic cylinder of 19 cm (inner diameter) and 21 cm in length (produced by Elektronika i Elektromedycyna, Poland, with certificate, European norms: EN ISO 9001 and EN ISO 13485) (Fig. 3a). A detailed description of the apparatus and the distribution of the magnetic field was provided in a previous report [34]. This applicator allows producing a variable, homogeneous, sine-wave alternating magnetic field with 50 Hz of frequency and magnetic flux densities ranging from 0.1 to 8 mT. The distribution of the magnetic field along the main axis is shown in Fig. 3c, d, e, and f. The polarization of the field was vertical (field lines perpendicular to the bottom plane of the animal’s cage) and the coil was able to ensure the maximum homogeneity (within 8 %) in a central cylindrical area (*r* = 7.5 cm, h = 7.5 cm), centered in the middle zone of the coil where the glass chamber containing the insects was placed (Fig. 3b). A nonmagnetic support was used to place the glass chamber in the coil. A sham apparatus was also constructed, with the same size and temperature but lacking a wound coil. Housed in a glass chamber (15 cm × 7.5 cm in diameter) to enable free movement, the insects were subsequently placed inside the exposure apparatus and then exposed to the magnetic field or to the sham exposure. The coil was located in an isolated room (with controlled light and temperature T = 23 ± 1 °C). The control group of the insects was handled in an identical manner without being exposed to any electromagnetic field. The magnetic field intensities were controlled before each experiment in a few different points of glass chamber by using a digital Gaussmeter Model GM2, AlphaLab, Inc. (to ensure stable values of the magnetic flux density during the course of the experiment).

### Statistical analysis

The data were analyzed using Stat SPSS software (IBM Corp. Released 2013. IBM SPSS Statistics for Windows, version 22.0. Armonk, USA). The results were expressed as means ± SEM and the comparison of several data groups was made using Kruskal-Wallis test. The differences between groups were tested by Mann-Whitney post-hoc tests. A value of p < 0.05 was considered to be significant (*p < 0.05; **p < 0.001; ***p < 0.0001).

## Results

### General description

Bioelectrical activity of the cockroach nerve cord was recorded using extracellular electrodes. The same protocol of recordings (Fig. 2) was applied to the cercal nerve (peripheral nervous system) and to the connective nerve leaving the terminal abdominal ganglion (central nervous system). Each record consisted of: (1) resting (spontaneous) activity – usually very low, (2) response to mechanical stimulation of the cercus – usually well defined through time, (3) again resting activity – generally very low. Examples of the recordings are presented in Fig. 1a, b, and c. Stimulus is marked with an arrow – it was the moment when a generator signal was sent to the loudspeaker membrane. The effect of the LqhαIT toxin was estimated on the basis of the size of the response to the stimulus. The size of the response was calculated as “response surface” meaning that the time of the response was multiplied by all bioelectric signals that appeared during this time. The duration of the response was very repetitive and the time of the response selected at the beginning was applied to all recordings in the experiment. It is important to note that (1) the same stimulus always generated a smaller response in the connective nerve than it did in the cercal nerve and (2) that the answer delay was bigger after a synaptic transmission (Fig. 1a and b). An increased stimulus induced a larger response and with a shorter delay (Fig. 1c).

The recordings of the bioelectrical activity in each experiment were made every ten seconds; ten records have been made in one set of series. After four series of control recordings, the exposure to 0.7 mT ELF-EMF was carried out for seven minutes. After five minutes of the ELF-EMF exposure, the LqhαIT toxin (5 × 10^−8^ M) was added – as described in the Methods section. The bioelectrical activity was recorded again after the end of the ELF-EMF exposure. Control experiments were performed according to the corresponding protocol (Fig. 2); however, without exposure to ELF-EMF.

### The effects of the LqhαIT toxin on the cercal nerve

**Fig. 4:**
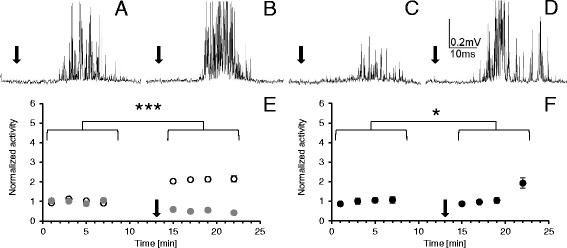
The effects of the LqhαIT toxin on cockroach cercal nerve activity and their modification by ELF-EMF exposure. The extracellular recordings of the cercal nerve activity as a response to the circus mechanostimulation (marked by black arrow) in: (**a**) control, (**b**) after the toxin (5 × 10^−8^ M) application, (**c**) after the exposure to ELF-EMF – 0.7 mT, (**d**) after the LqhαIT toxin (5 × 10^−8^ M) application and exposure to the ELF-EMF – 0.7 mT. (**e**) Normalized bioelectrical activity of the cercal nerve during the experiment in the control conditions (white and grey circles), after toxin application (white circles) and after exposure to ELF-EMF (grey circles). (**f**) Normalized activity of the cercal nerve activity after the LqhαIT toxin application and the exposure in the ELF-EMF at the same time. On **e** and **f**: arrow corresponds to the application of the toxin. Note: the scheme of the experimental protocol is shown on Fig. 2. Data are presented as mean values ± standard errors; standard errors are in most cases inside the symbols, *n* = 15

Under controlled conditions, the size of the response to the mechanostimulation of the cercus was stable for at least one hour; however, there was a relatively large variation in the bioelectrical activity and in the stimulus threshold among preparations. This prompted us to measure and compare all values with activity estimated during the first minute of the experiment and to present it in a normalized form. Mean value of all control records was established as 1. The application of the LqhαIT toxin (5 × 10^−8^ M) already in the 3^rd^ minute clearly increased discharge frequency in the cercal nerve (Fig. 4a, b, e) and its value almost doubled (with statistical significance p < 0.0001) and stabilized during the next eight minutes. After the control recordings, the next group of preparations was exposed to 0.7 mT ELF-EMF for seven minutes. The bioelectrical activity was recorded immediately after ending the exposure and it appeared to be about 50 % lower than in the control conditions (Fig. 4a, c, e). The application of the toxin during the exposure to the electromagnetic field elevated the size of the response when compared to the preparations only exposed to the ELF-EMF (Fig. 4d, f). The value of the response was stabilized for five minutes at the same level as in the control recordings, and it was still much lower than in the case when the toxin was applied without the ELF-EMF exposure. Only at the end of the experiment, the bioelectrical activity was slightly higher.

### The effects of the toxin LqhαIT on the connective nerve

**Fig. 5:**
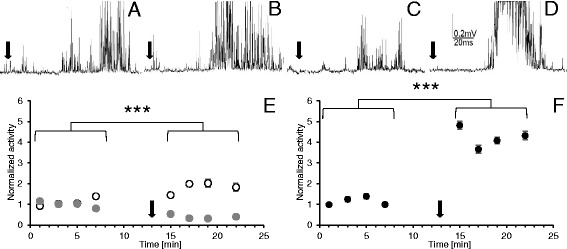
Effects of the LqhαIT toxin on the cockroach connective nerve activity and their modification by the ELF-EMF exposure. The extracellular recordings of the connective nerve activity as a response to the circus mechanostimulation (marked by black arrow) in: (**a**) control, (**b**) after the toxin (5 × 10^−8^ M) application, (**c**) after the exposure to ELF-EMF – 0.7 mT, (**d**) after the toxin (5 × 10^−8^ M) administration and exposure to ELF-EMF – 0.7 mT. (**e**) The normalized activity of the connective nerve during the experiment in the control conditions (white and grey circles), after the toxin application (white circles) and after exposure to ELF-EMF (grey circles). (**f**) The normalized connective nerve activity after the toxin application and exposure to ELF-EMF at the same time. On **e** and **f** arrow corresponds to the application of the toxin. Note: the scheme of the experimental protocol is shown on Fig. 2. Data are presented as mean values ± standard errors (S.E. are in most cases inside the symbols, *n* = 15)

The response to the mechanostimulation of the cercus recorded from the connective nerve was more dispersed in time compared to the response recorded from the cercal nerve (Fig. 4a and Fig. 5a). Application of the toxin induced an effect similar to that on the cercal nerve. Discharge frequency doubled five minutes after the toxin application and was stable during the following minutes (Fig. 5a, b, e). Increase in the size of the response induced by the toxin was statistically significant with p < 0.0001. The effect of the ELF-EMF exposure on the connective activity was similar to that observed on the cercal nerve. The response to the mechanostimulation was evidently reduced, its lowest value reached only 32.2 % of the control (Fig. 5c, e). The application of LqhαIT (5 × 10^−8^ M) rapidly produced a large increase in the connective nerve bioelectrical activity. Three minutes after the toxin administration, the size of the response was more than four times higher than that observed in control (with a high statistical significance p < 0.0001). During the next minute, the response decreased; however, it remained on a much higher level than that observed in the control. Later, the changes were smaller but the activity increased slightly again.

**Fig. 6:**
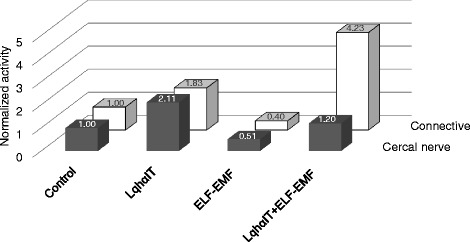
The influence of ELF-EMF exposure on LqhαIT toxin induced “electrophysiological activity” – a summary. Changes in the average normalized activity of the cercal nerve (black columns) and the connective nerve (white columns) after: LqhαIT toxin (5 × 10^−8^ M) application, ELF-EMF (0.7 mT) exposure, and toxin application and ELF-EMF exposure at the same time. Standard errors are omitted for clarity but are indicated at Figs. 4 and 5

The aim of our study was to determine whether the exposure to 50 Hz electromagnetic field changes the effect of the anti-insect scorpion alpha toxin on the peripheral and the central nervous system of a cockroach. To clarify our conclusions, we have prepared Fig. 6 that summarizes the results. We can outline that (1) the effect of the LqhαIT toxin on the cercal nerve is similar to the toxin effect on the connective nerve, (2) the influence of the ELF-EMF on the bioelectrical activity is similar in the cercal nerve and the connective nerve levels, (3) the exposure to ELF-EMF decreases the toxin effect on the cercal nerve activity and increases its influnce on the connective nerve.

### Effects of the toxin LqhαIT on cockroaches

The second part of our study was performed on cockroaches *in vivo* and the state of insect paralysis induced by LqhαIT (10^−8^ M) was observed. The paralysis was induced by overexcitation of terminal branches of motoneurons and subsequently muscle convulsions.

**Fig. 7:**
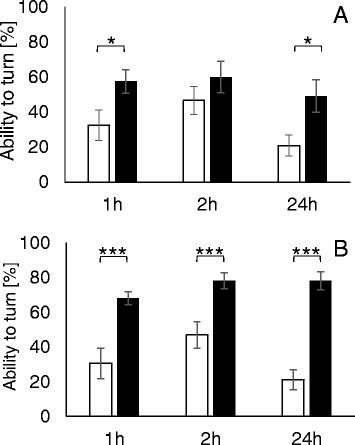
The influence of ELF-EMF on the insect paralysis induced by the LqhαIT toxin injection -experiments *in vivo.* Bars represent the level of insect paralysis; it is expressed using a scale from 0 to 4, in which 4 is the full ability of insects to turn back from the ventral to the dorsal side, 1 means that insects are completly unable to turn back to the normal position. Time: 1, 2 and 24 h after the toxin administration and the start of ELF-EMF exposure. (**a**) LqhαIT (10^−8^ M) effect (white column) and LqhαIT effect under exposure to ELF-EMF (0.7 mT) (black column). (**b**) LqhαIT (10^−8^ M) effect (white column) and LqhαIT effect under exposure to the ELF-EMF (7 mT) (black column)

The ability of every single cockroach to turn from the dorsal to the normal position was assessed and expressed on scale from 0 to 4 – as indicated in the Methods section. All control insects were able to turn back from the dorsal to the ventral side immediately. The dose of the toxin applied was sublethal and caused a 70 % reduction (to 1.22 value on our scale) in the insect rotation capacity one hour after toxin application (Fig. 7a, b). After the second hour, a smaller number of insects was paralyzed, which indicated a small reversibility of toxin effects. After 24 h, the ability of cockroaches to turn back to normal position was only about 20 %. However, the differences between the described values were not statistically significant.

The next groups of insects were exposed to 50 Hz electromagnetic field of 0.7 mT intensity (Fig. 7a) and of 7 mT intensity (Fig. 7b). Notably, ELF-EMF exposure decreased toxicity in all experiments. The average insect ability to turn back increased following ELF-EMF exposure (0.7 mT) from 30 to 55 % (from 1.2 to 2.2 on our scale) in the first hour after the toxin administration and remained similar after 2 and 24 h (Fig. 7a). The differences after 24 h between the toxin effect on exposed and nonexposed cockroachs were significant with p < 0.05. A ten-time higher electromagnetic field intensity (7 mT) had much stronger effects. The insect ability to turn back to the normal position reached 67 % (2.7 on our scale; p < 0.0001) and 75 % in the first and second hour, respectively, and stayed at a similar level after 24 h from the toxin application (Fig. 7b). These results indicate the protective influence of ELF-EMF exposure against the toxic effects of LqhαIT, which was larger in a higher magnetic ELF-EMF intensity.

## Discussion

The aim of our study was to estimate the influence of 50 Hz electromagnetic field on the efficiency of the anti-insect alpha scorpion toxin (LqhαIT) on the cockroach, *Periplaneta americana*. This insect was used as a model in numerous neurophysiological and pharmacological studies [17, 35]. The toxin LqhαIT and its recombinant forms are the most investigated toxins in the group of anti-insect scorpion alpha toxins [13, 16, 36] and they have been tested in detail on cockroach preparations [13, 17]. The toxin LqhαIT, like other scorpion alpha toxins, inhibits a fast inactivation of a sodium channel and increases the channel open time leading to a higher activity and depolarization of presynaptic terminals, causing, in turn (1) an increase of the calcium channel opening, (2) an increase in the calcium influx and the elevation of its intracellular level, (3) a hyper-release of different neurotransmitters (e.g. acetylcholine and glutamate), (4) an overstimulation of receptors corresponding to neurotransmitters, (5) an activation of diverse cellular responses *via* different signaling pathways, and (6) an overstimulation of muscle end-plates resulting in convulsions and/or a complete paralysis [18, 37, 38].

### Effects of ELF-EMF on the electrophysiological properties of peripheral nerve (the cercal nerve) in cochroach

The effects of the electromagentic field exposure on the toxin efficiency may be discussed on different levels of the organism organisation. However, in our electrophysiological experiments, we wanted to assess the effect of the ELF-EMF (1) directly on nerves with simple cable properties and (2) to estimate its effect on the synaptic transmission level. It was important to observe the effect of the toxin quickly and right away, so that the influence of the ELF-EMF on the interaction of the toxin with membrane sodium channels could be estimated.

Anatomically, an intact central nervous system of insects is almost impermeable to scorpion anti-insect toxins; however, its peripheral regions including cercal nerves and the terminal branches of motor nerves are relatively well accessible [39, 40]. An immediate increase of the cercal nerve bioelectrical activity was observed after LqhαIT application, which indicates good accessibility of toxins to the cercal axonal membrane. This undoubtedly results from the direct effect of the toxin on membrane permeability for sodium ions. Exposure to a weak ELF-EMF (0.7 mT) reduced discharges recorded from the cercal axons. This clearly demonstrates that the function of the mechanosensory neurons was perturbed by the ELF-EMF influence. We consider these effects as the additional evidence that electromagnetic fields can modify basic electrical neuronal membrane properties.

In the case of the application of the LqhαIT toxin under the exposure to ELF-EMF, the induced effects were smaller. Suppression of the toxin effect was higher than the summation of toxin and ELF-EMF effects together (Fig. 6). Dissociation binding assays on the anti-insect scorpion toxin AaHIT showed that the tolerance to the toxin is closely correlated with the stability of the toxin-receptor complex [40]. The obtained results suggest that an electromagnetic field decreases the interaction (binding affinity) of the alpha toxin with the sodium channel. This fits well with the hypothesis postulated by Saunders and Jefferys [7] that electromagnetic fields exert direct effects on the electric dipole voltage sensor in voltage gated ionic channels. Further studies are necessary to clarify the mechanism of the ELF-EMF influence on the toxin binding to receptor site 3 on the sodium channel using, for example, molecular dynamics simulations.

### Effects of ELF-EMF on the electrophysiological properties of the connective (central) nerve

The recordings carried out on the nerve cord connective nerve leaving the last abdominal ganglion showed that the toxin LqhαIT almost doubled the discharge frequency – similarly to the one observed in the cercal nerve (Fig. 6). During the analysis, we needed to consider low accessibility of the toxin to the inside of the ganglion where synapses are localized. As it is shown in Fig. 1a, b the response to the mechanostimulation is higher at the level of the cercal nerve than the one recorded at the connective nerve. Considering that, we suggest that the effect of the toxin was amplified when information was passed by synapses (Fig. 5a, b). It should be noted that later, the activity of the connective nerve was decreased (not shown), most likely due to activation of negative feedback mechanisms, as has been previously described in the insect central nervous system [41, 42].

The connective nerve bioelectrical activity decreased under the exposure to the electromagnetic field. Similar results were obtained by other authors. Sinusoidal magnetic fields (50 Hz) lower than 0.21 mT inhibited spontaneous bioelectrical activity in snail neurons [2] and in isolated neurons from dorsal root ganglia [43]. In our unpublished results (J. Wyszkowska), we observed the decline of the spontaneous activity of cockroach neurosecretory dorsal unapired median neurons under the exposure to 0.7 mT ELF-EMF. The suppressive effect of ELF-EMF could be explained by higher activation of calcium-dependent potassium channels (K_Ca_) [2, 43]. A similar explanation might be applied to our research. The effect of ELF-EMF exposure observed in our experiments may be mediated by the increase in intracellular calcium concentration. A higher [Ca^2+^]_i_ may shift the voltage dependence of K_Ca_ channel activation to more negative membrane potentials. A faster activation of K_Ca_ causes hyperpolarization of the membrane, limits calcium entry and, subsequently, reduces transmitter release [44]. Finally, the activity of the connective nerve under the exposure to the ELF-EMF may be dependent on (1) the influence of an electromagnetic field on membrane properties of cercal axons, (2) an increase of [Ca^2+^]_i_ in presynaptic terminals and higher release of acetylcholine, (3) limiting effects of K_Ca_ on presynaptic terminal activity. Unexpectedly, the application of LqhαIT toxin during the exposure to ELF-EMF induced a sudden increase (almost four fold) of the connective activity. A large toxin-induced presynaptic activity and a high level of Ca^2+^ influx amplified by the exposure to the ELF-EMF could eliminate the compensatory participation of K_Ca_ in synaptic function. Moreover, the LqhαIT could increase the releasing of different neurotransmitters such as octopamine, which, for example, increases the excitability of giant interneurons and response to wind stimulation of the cerci [45–47]. Subsequently, the decrease and again the increase of bioelectric activity of the connective nerve could have resulted from the function of the regulatory feedback by muscarinic receptors, as mentioned earlier. In this paper, we evidenced that the exposure to the ELF-EMF modifies the LqhαIT toxin effect on bioelectrical activity of the insect peripheral and central nervous system. Amplification of the electromagnetic field effects on the synaptic level was visible in our experiments; however, it needs further study.

### Effects of ELF-EMF on the entire cockroach

Biotests were performed with a sublethal dose of LqhαIT (10^−8^ M) that induced paralysis in less than 50 % of the insects. Exposure to ELF-EMF decreased the toxicity of LqhαIT. Cockroach capability to turn back from the dorsal side to the normal position was much higher when they were influenced by an electromagnetic field. This capability to turn back was “dose-dependent”, meaning that with a higher ELF-EMF intensity, the paralysis was lower until the end of the observations. First observations of the insects were carried out one hour after toxin administration and the beginning of ELF-EMF exposure. During this time, many physiological compensatory reactions may have occurred at the organism level, such as turning on detoxification mechanisms that have probably been amplified by the ELF-EMF exposure.

Degradation of the toxin and its elimination from the insect body may be modified by different factors (e.g. metabolic rate), as in the case of all detoxification processes. Detoxification is more rapid in organisms with higher metabolic rate. There are several reports demonstrating that the exposure to an electromagnetic field increases the activity of cells [5, 12].

The main stress hormone in insects is octopamine, which is an analog of vertebrate norepinephryne [47]. Wyszkowska *et al.* [48] demonstrated that exposure to ELF-EMF (7 mT) induces an increase in locomotor activity in cockroaches. Such effect was suppressed by phentolamine, the blocker of octopamenergic receptors. Moreover, the concentration of octopamine in cockroach hemolimph was higher after the exposure to an electromagnetic field than in control (J. Wyszkowska, unpublished results). Together, these strongly suggest that changes in the octopamine level may be a key component underlying the influence of the ELF-EMF on insect organism.

We demonstrated that the exposure to 50 Hz electromagnetic field changed the efficiency of the scorpion alpha toxin LqhαIT on a cockroach and its nervous system. To the best of our knowledge, this is the first report describing the effects of the ELF-EMF on the activity of natural neurotoxins. We assume that our rather simple experimental model with the application of a toxin whose binding ability may be modified by charge orientation and redistribution will help to better define the “primary and secondary” mechanisms of ELF-EMF influence on organisms.

## Conclusions

The exposure to 50 Hz electromagnetic field modified the efficiency of the anti-insect scorpion alpha toxin LqhαIT on a cockroach and its nervous system. The exposure to ELF-EMF slightly decreased the toxin effect on the cercal nerve activity and largely increased its influence on the connective nerve activity. We suggest that such phenomena are the effects of a direct influence of an electromagnetic field on excitable membranes and on synaptic transmission. In biotests, the observed decrease of LqhαIT toxicity may be explained by the indirect ELF-EMF influence on the insect metabolic rate or intracellular signaling. We argue that LqhαIT toxin may serve as an excellent tool in distinguishing between the primary and the secondary effects of ELF-EMF. We intend to use this toxin as a starting point for experiments to further examine the ramification of a long-term exposure in elelctromagnetic field on biological entities.

## Abbreviations

ELF-EMF: Extremely low-frequency electromagnetic field
LqhαIT: Recombinant anti-insect alpha toxin from *Leiurus quinquestriatus hebraeus* scorpion
K_Ca_: Calcium-dependent potassium channels
